# Ordinary varieties with trivial canonical bundle are not uniruled

**DOI:** 10.1007/s00208-021-02165-y

**Published:** 2021-06-09

**Authors:** Zsolt Patakfalvi, Maciej Zdanowicz

**Affiliations:** Chair of Algebraic Geometry, École Polytechnique Fédérale de Lausanne, MA C3 625 (Bâtiment MA), Station 8, 1015 Lausanne, Switzerland

**Keywords:** Primary 14G17, Secondary 14M17, 14M25, 14J45

## Abstract

We prove that smooth, projective, *K*-trivial, weakly ordinary varieties over a perfect field of characteristic $p>0$ are not geometrically uniruled. We also show a singular version of our theorem, which is sharp in multiple aspects. Our work, together with Langer’s results, implies that varieties of the above type have strongly semistable tangent bundles with respect to every polarization.

## Introduction

We work over a perfect field *k* of characteristic $p>0$.

Consider a smooth projective *K*-trivial variety *X* of dimension *d* defined over *k*, where *K*-trivial means that $K_X \sim 0$. Our main theorem connects cohomological properties of *X*, classically believed to measure how similar the variety is to its characteristic zero analogues, with standard geometric properties related to existence of rational subvarieties. The smooth case of our result is:

### Theorem 1.1

(Theorem 5.6) Smooth case: If *X* is a smooth weakly ordinary projective variety over *k* satisfying $K_X \sim 0$, then *X* is not geometrically uniruled.

We note straight away that Theorem 1.1 actually works more generally for $K_X \equiv 0$ by replacing weak ordinarity with global *F*-splitting, see Theorem 5.6, which allows for mild singularities on *X*. Before passing to the description of our approach let us first explain the motivation and provide a landscape of relevant notions and available techniques. As mentioned above the geometric properties which we will be concerned with mostly relate to existence of rational subvarieties or rational parametrizations. The most important examples of such properties are:

- (unirat) *X* is *unirational*: it admits a generically finite dominant rational map $\mathbb {P}^n \dashrightarrow X$,
- (unirul) *X* is *uniruled*: it admits a generically finite dominant rational map $\mathbb {P}^1 \times Z \dashrightarrow X$,
- (rcc) *X* is *rationally chain connected*: two general points of *X* can be connected by a chain of rational curves.

We emphasize that those properties never happen for *K*-trivial varieties in characteristic zero, and only hold over loci that are not dense in moduli in characteristic $p>0$. Let us now focus on the properties considered on the cohomological side. The first three of the following are genericity conditions that typically hold over dense open sets of moduli spaces. The last one typically holds on complements of such open dense sets and was used by Shioda in his approach to the two-dimensional predecessor of our result (see Sect. 1.2 for some historical details).

- (w-ord) *X* is *weakly ordinary*: the action of the Frobenius morphism on $H^{d}(X, \mathcal {O}_X)$ is bijective,
- (ord) *X* is *ordinary*, see [6, Definition 7.2],
- (Witt) $$ H^d\left( X, W\mathcal {O}_{X, \mathbb {Q}} \right) \ne 0$$,
- (Shio) $H^2(X, \mathcal {O}_X) \ne 0$, but algebraic cycles span the entire $H^2_{{\mathrm{K}\acute{\mathrm{e}}\mathrm{t}}}\left( X_{\overline{k}}, \mathbb {Q}_l(1)\right)$ (Shioda supersingular).

Many important results and conjectures of the area concern connections between the above notions. In the following diagram, where the top row contains the geometric and the bottom row the cohomological notions, we portray some of what is known for : 1.1a It is particularly interesting to connect the cohomological and the geometric side. In this spirit, the topic of our main result Theorem 1.1, also shown by the left vertical arrow on diagram (1.1a), is about relation between coherent cohomology invariants, precisely Frobenius action on the top cohomology of the structure sheaf, and uniruledness. For more historical comments and additional information on diagram (1.1a), we again refer to Sect. 1.2. We now remark that to the best of our knowledge Theorem 1.1 is the first result in arbitrary dimension involving only coherent cohomology. In particular, the cohomological condition of Theorem 1.1 is probably the first one in arbitrary dimension that can be verified computationally. In this sense Theorem 1.1 can be regarded as a version of [15] (which is of cycle-theoretic nature) with a cohomological condition that can be checked effectively. In another direction Theorem 1.1 can be also regarded as a version of [42] for general *K*-trivial varieties, as opposed to complete intersections. Additionally, it can be also regarded, via diagram (1.1a), as a generalization of the K3 surface result [46].

The classical works [2, 46], shown by the right vertical arrow of (1.1a) and reviewed in Sect. 1.2, are very specific to K3 surfaces. Hence, our approach is more similar in spirit to the diagonal arrow of (1.1a): we use Witt cohomology and *p*-adic cohomology invariants. However, as mentioned already in the bottom row of the diagram (1.1a), the direct connection between weak ordinarity and the non-vanishing of $H^d \left( X, W\mathcal {O}_{X, \mathbb {Q}} \right)$ works only if $H^{d-1}(X, \mathcal {O}_X) \ne 0$ (though, in [24, Theorem 3.2.1] Joshi and Rajan proved finiteness of $H^d (X, W\mathcal {O}_{X})$ over *W*(*k*) in our situation). As for example *X* could have non-trivial Albanese morphism, in which case $H^{d-1}(X, \mathcal {O}_X) \cong H^1(X, \mathcal {O}_X) \ne 0$ would hold, there is no hope to apply this connection directly to *X*. We solve this issue by passing to the geometric generic fiber of the maximal rationally chain connected fibration (MRCC fibration for short). In particular, most of the article is about finding a singularity class which satisfies both of the following:

- the geometric generic fiber of the MRCC fibration has these types of singularities, and
- we are able to show the non uniruledness in the presence of this class of singularities.

In other words, it is inherent for our methods (see Sect. 1.1 for details) that we prove a singular version of Theorem 1.1 too:

### Theorem 1.2

(Theorem 5.6) Singular case: Let *X* be a normal, $S_3$, projective, globally *F*-split variety over *k* with $W\mathcal {O}$-rational singularities and with $K_X\sim 0$. Then *X* is not geometrically uniruled.

In Theorem 5.6, we use the condition of *F*-splitting which is equivalent to weak ordinarity if $K_X$ is actually trivial (see Proposition 2.6 or [35] and [36] for classical accounts). This property is better suited for the purpose of our proof—it naturally descends to the general fibers of fibrations. Unfortunately, we were not able to prove that same statement for $W\mathcal {O}$-rationality, which explains the presence of *p*-adic cohomology in the argument. More precisely, using proper base change, to the best of our knowledge unavailable for $W\mathcal {O}$-cohomology, we managed to prove that the general fiber of a fibration of a $W\mathcal {O}$-rational variety admits a quasi-resolution preserving *p*-adic cohomology (Proposition 3.22, see Definition 3.6 for the definition of a quasi-resolution). This leaves a natural question:

### Question 1.3

Let *X* be a $W\mathcal {O}$-rational variety. Assume that $f :X \rightarrow S$ is a fibration with geometric generic fibre reduced. Is the geometric generic fiber also $W\mathcal {O}$-rational?

We note that a characteristic zero analogue of the above statement, involving the notion of rationality, can in fact be proven using a simple base change argument.

### Remark 1.4

The assumption of $W\mathcal {O}$-rationality in Theorem 1.2 is satisfied in a few important special cases. For example, if *X* is rational, that is, there exists a resolution of singularities $f :Y \rightarrow X$ such that $Rf_*\mathcal {O}_Y = \mathcal {O}_X$, or if *X* is a klt threefold (see [18, Theorem 1.4]).

We finish this part of the introduction by noting that by the previous work of the authors [39], a Beauville–Bogomolov type decomposition holds for *K*-trivial weakly ordinary varieties. We remark that in characteristic zero the full decomposition theorem relies on stability properties of the tangent bundle. In this spirit, using recent result of Langer [29, Corollary 3.3] we obtain the following:

### Corollary 1.5

If *X* is a smooth projective weakly ordinary (resp. *F*-split) variety with $K_X \sim 0$ (resp. $K_X \equiv 0$), then the tangent sheaf $\mathcal {T}_X$ is strongly *H*-semistable for every ample divisor *H* on *X*.

### Strategy of the proof

In this section, we present a brief description of the proof of our main theorem. Let *X* be as in the statement of Theorem 1.1, and set $d := \dim X$. In order to explain the argument let us first list two basic observations:

1. if *X* is uniruled, then both $H^d(X, W \mathcal {O}_{X,\mathbb {Q}})$ and $H^d_{{\mathrm{K}\acute{\mathrm{e}}\mathrm{t}}}(X,\mathbb {Q}_p)$ vanish by Proposition 4.7,
2. by Serre duality, as $K_X \sim 0$, we have $H^d(X, \mathcal {O}_X) \cong H^0(X, \mathcal {O}_X) =k$.

These facts motivate the initial idea to deduce that point (2), together with the weak ordinarity of *X*, implies that $H^d(X,W\mathcal {O}_{X,\mathbb {Q}}) \ne 0$, yielding a contradiction with point (1). The natural argument implementing this idea could be based on the series of long exact sequence of cohomology

$$\begin{aligned} \ldots \rightarrow H^i(X, \mathcal {O}_X) \rightarrow H^i(X,W_{n+1}\mathcal {O}_X) \rightarrow H^i(X,W_n\mathcal {O}_X) \rightarrow \ldots \end{aligned}$$

associated to short exact sequences of finite length Witt vector sheaves, where *V* is the Verschiebung homomorphism:We would expect that this way Witt vector cohomology in degree *i* is constructed from coherent cohomology in the same degree. The main point of our approach is that this argument does not always work. There are natural obstructions given by the edge maps in the above long exact sequences of cohomology. These obstructions are called Bockstein operations, and were analyzed thoroughly by Mumford [37, Chapter 27] in relation to the phenomena of non-reducedness of the Picard scheme in characteristic *p* geometry. It turns out that already in dimension two the relevant obstructions could actually be non-zero. For instance, every non-classical ordinary Enriques surface *S* in characteristic two is weakly ordinary and it satisfies the conditions: $K_S \sim 0$ and $H^2(S,\mathcal {O}_S) \ne 0$, but $H^2(S,W\mathcal {O}_{S,\mathbb {Q}}) = 0$, see Example 5.9.

We note that, weakly ordinarity is also essential. In fact, even under the assumption that all Bockstein operations vanish, the top Witt vector cohomology $H^d(X,W\mathcal {O}_{X,\mathbb {Q}})$ could vanish. However, if $H^{d-1}(X, \mathcal {O}_X)=0$, through the vanishing of Bockstein operations, weak ordinarity implies that the integral Witt vector cohomology $H^d(X,W\mathcal {O}_X)$ is in fact a non-zero *p*-torsion free *W*(*k*)-module which yields the necessary non-vanishing after localization by $\mathbb {Q}$, see Proposition 4.1. Since *X* is ordinary, we also show that the *F*-isocrystal $H^d(X,W\mathcal {O}_{X,\mathbb {Q}})$ is generated by its Frobenius fixed points, which implies that $H^d_{{\mathrm{K}\acute{\mathrm{e}}\mathrm{t}}}(X,\mathbb {Q}_p)$ does not vanish either (also Proposition 4.1). .

Summing up, if we manage to put ourselves in the situation where $H^{d-1}(X, \mathcal {O}_X) = 0$, while keeping enough assumptions so that the above argument still holds, then we get a contradiction. Indeed, in this case we have that $H^d(X, W \mathcal {O}_{X,\mathbb {Q}})$ and $H^d_{{\mathrm{K}\acute{\mathrm{e}}\mathrm{t}}}(X,\mathbb {Q}_p)$ vanish by first paragraph, and at the same time they are non-zero by the reasoning after the first paragraph. Below, we explain how we manage to conduct a similar argument, however working only for $H^d_{{\mathrm{K}\acute{\mathrm{e}}\mathrm{t}}}(X,\mathbb {Q}_p)$.

The main tool will be the *maximal rationally chain connected* (abrv. MRCC) fibration. More specifically, we recall that Kollár’s theorem (see Theorem 5.2) states that every proper uniruled variety *X* admits an open subset $X^\circ$ along with a proper morphism $f :X^\circ \rightarrow S$ of positive relative dimension satisfying the properties:

- $$f_*\mathscr {O}_{X^\circ } = \mathscr {O}_S$$,
- general fiber of *f* is rationally chain connected.

and *f* is maximal with respect to those properties (see Sect. 5.1 for the details). Intuitively, the maximal rationally chain connected subvarieties of every uniruled variety can be exhibited as fibers of a proper fibration. We note that using the adjunction the positivity properties of the canonical divisor are preserved when passing from the total space to the fibers of MRCC fibration given the singularities are controlled.

We now claim that we may replace *X* by the general fiber of its MRCC fibration possibly introducing mild singularities but gaining rational chain connectedness (see Example 5.10 for an example of a variety that we need to rule out as a fiber). First, in our situation the assumption of weak ordinarity is equivalent to *F*-splitting which is inherited by general fibers of morphisms [17, Cor 2.5]. Then in this situation normality is also inherited by [46, Thm 1.3.(3)]. As already mentioned above, in Proposition 3.22 we also show that *X*, while not necessarily $W\mathcal {O}$-rational, still admits a quasi-resolution $Y \rightarrow X$ which does not change *p*-adic cohomology. Now, by a fundamental group argument using weak ordinarity and the Artin–Schreier sequence (see Lemma 5.3 and Theorem 5.4) one sees that $H^{d-1}(X,\mathcal {O}_X) = H^1(X,\mathcal {O}_X)^{\vee } = 0$, after possibly replacing *X* with its étale cover, and hence $H^d\left( X,W\mathcal {O}_{X,\mathbb {Q}}\right)$ and $H^d_{{\mathrm{K}\acute{\mathrm{e}}\mathrm{t}}}(X,\mathbb {Q}_p)$ are non-zero. The total space *Y* of the postulated quasi-resolution is at least uniruled and hence satisfies the vanishing $H^d_{{\mathrm{K}\acute{\mathrm{e}}\mathrm{t}}}(Y,\mathbb {Q}_p)$ by Proposition 4.7. This gives a contradiction and hence finishes the proof.

### Historical remarks about the K3 surface case

We note that in characteristic $p>0$ geometry there exist unirational hypersurfaces in $\mathbb {P}^3$ of every Kodaira dimension. For instance, in [46] Shioda provides a remarkable computational proof of the fact that Fermat hypersurfaces

$$\begin{aligned}&X_n = \{x^n + y^n + z^n + u^n = 0\} \subset \mathbb {P}^3_k, \\&\quad k \text { is an algebraically closed field of characteristic } p>0 \end{aligned}$$

are *unirational* if there exists a positive integer *e* such that $p^e \equiv -1\pmod {n}$. In the same paper, Shioda deduces that under the same condition on *p* and *n*, the second cohomology group of surfaces $X_n$, in the $\ell$-adic or crystalline sense, is generated by algebraic cycles. Consequently, the Picard rank $\rho _{X_n}$ is maximal possible, equal to the second Betti number. In general surfaces satisfying this condition are called *Shioda supersingular*.

In the special case when $n = 4$ and for $p \equiv 3\pmod {4}$, by adjunction formula, the quartic surface $X_4$ is a Shioda supersingular K3 surface. In the paper [2], Artin investigated the necessary condition for a K3 surface to be Shioda supersingular using Brauer groups. In particular, he proved that for a K3 surface *X* the Picard rank satisfies the inequality $\rho _X \le 22 - 2h_X$, if the height $h_X$ of the formal Brauer group $\Phi _X$ is finite. Consequently, no K3 surfaces with finite $h_X$ could be Shioda supersingular, and hence unirational. The number $h_X$ turns out to be equal to the rank over *W*(*k*) of the module $H^2(X,W\mathcal {O}_X)$, where $W\mathcal {O}_X$ is the sheaf of Witt vectors of $\mathcal {O}_X$, the Dieudonné module associated to $\Phi _X$ [1, Proposition 2.13]. By a simple computation (see Proposition 4.1) one observes that $h_X$ is equal to one in the particular case when the Frobenius action on the one-dimensional top cohomology group $H^2(X,\mathcal {O}_X)$ is bijective, that is, the surface *X* is weakly ordinary. On the other hand, as a consequence of Tate conjectures for K3 surfaces $h_X$ being infinite is in fact equivalent to *X* being Shioda supersingular [8]. Shioda also conjectures that in this case *X* is unirational, which has been an active area of research in the past few decades (see, e.g., [7, 31, 33, 41, 47]).

## General preliminaries

In this preliminary section we gather some definitions and results required in our considerations.

### Notation and conventions

In the present paper, unless stated otherwise, all schemes are defined over a perfect field *k* of characteristic $p>0$. By $W_n(k)$ (resp. *W*(*k*)) we denote the ring of Witt vectors of length *n* (resp. infinite length) and by *K* the fraction field of the ring *W*(*k*). A *variety* is a separated integral scheme of finite type defined over *k*. We say that a morphism $f :X \rightarrow Y$ is a *fibration* if it is proper and satisfies $f_*\mathcal {O}_X = \mathcal {O}_{Y}$.

### *F*-splitting and ordinarity

Let $f :X \rightarrow S$ be a morphism of schemes over a perfect field *k*. We recall that the *e*-th relative Frobenius $F^e_{X/S}$ morphism is defined by the following commutative diagram:

#### Definition 2.1

We say that *f* is *globally* *F-split* if there exists a splitting of the natural map $\mathcal {O}_{X^e_S} \rightarrow F^e_{X/S,*}\mathcal {O}_X$, as a homomorphism of $\mathcal {O}_{X^e_S}$-modules. In particular, a scheme *X* defined in characteristic *p* is *globally* *F-split* if the natural map $X \rightarrow {{\,\mathrm{Spec}\,}}(\mathbb {F}_p)$ is globally *F*-split (equivalently, the natural homomorphism $\mathcal {O}_X \rightarrow F_*\mathcal {O}_X$ is globally split).

Suppose $f :X \rightarrow S$ is a globally *F*-split morphism over *k*, and let $T \rightarrow S$ be a morphism. Taking a base change of a splitting we see that $f_T :X_T \rightarrow T$ is also globally *F*-split. In particular, fibres of a globally *F*-split morphism are globally *F*-split. We now recall the following result. In the proof, we adopt a few definitions included in [48]. In particular, we say that a $\mathbb {Q}$-divisor $\Delta$ is $\mathbb {Z}_{(p)}$-Weil (resp. $\mathbb {Z}_{(p)}$-Cartier) if there exists number *N* coprime to *p* such that $N \cdot \Delta$ is Weil (resp. Cartier). We note that such conditions can always by exhibited using a number of the form $p^f - 1$, for $f \in \mathbb {N}$.

#### Proposition 2.2

Let *X* be a globally *F*-split normal projective variety over *k* such that $K_X$ is $\mathbb {Q}$-Cartier with index prime-to-*p* and let $f :X \rightarrow S$ be a fibration. Then there exists a non-empty open subset $U \subset S$ such that $f_{|U} :X_U \rightarrow U$ is a globally *F*-split morphism. Moreover, there exists an open subset $V \subset X$ such that $V \rightarrow U$ is smooth and ${{\,\mathrm{codim}\,}}_X(X \setminus V) = 2$.

#### Proof

First, using the technique of [48, Proposition 3.12] we observe that there exists a divisor $\mathbb {Z}_{(p)}$-Cartier divisor $\Delta$ such that $(X,\Delta )$ is an *F*-split pair and $K_X + \Delta \sim _{\mathbb {Z}_{(p)}} 0$. Localizing the splitting of *X* at the generic point $\eta$ of *S* and then using the argument of [17, Proposition 2.6] for $D = 0$, we see that the geometric generic fibre $(X_{\overline{\eta }},\Delta _{\overline{\eta }})$ is *F*-split. By [38, Theorem B] the same condition holds for all geometric fibres in the neighbourhood of $\eta$. Using [49, Proposition 5.8 (3)], we may now assume that $X \rightarrow S$ is actually locally *F*-split (as defined in [49]) and therefore application of [49, Proposition 5.7] yields the desired result. $\square$

#### Corollary 2.3

Let *X* be a globally *F*-split normal projective variety over *k* such that $K_X$ is $\mathbb {Q}$-Cartier with index prime-to-*p*, and let $f :X \rightarrow S$ be a fibration. Then there exists a non-empty open subset $U \subset S$ such that for every geometric point $u \in U$ the fibre $X_u$ is globally *F*-split and normal.

In the following statement, we need notions of a dualizing complex $\omega ^\bullet _X$ and a dualizing module $\omega _X$ of a variety *X* defined over *k*. For a detailed description, we refer to [49, Tag 08XG] and [49, Tag 0A85]. We note that by definition $\omega _X \simeq \mathscr {H}^{-\dim X} \left( \omega _X^{\bullet } \right)$, and that for normal varieties $\omega _X$ is in fact an $S_2$ divisorial sheaf $\mathscr {O}_X(K_X)$ corresponding to the canonical divisor $K_X$.

#### Proposition 2.4

Let *X* be a projective equidimensional variety over *k*. Assume that *X* satisfies Serre’s condition $S_k$ and let $d = \dim X$. Then there is a functorial isomorphism $H^{d-i}(X,\omega _X) \simeq H^i(X,\mathscr {O}_X)^\vee$ for $i<k-1$. Moreover, there exists a natural surjective morphism $H^{d-k+1}(X,\omega _X) \rightarrow H^{k-1}(X,\mathscr {O}_X)^\vee$.

#### Proof

First, we observe that duality theory yields a functorial isomorphism ${{\,\mathrm{Ext}\,}}^{-i}(\mathscr {F},\omega ^\bullet _X) \simeq H^i(X,\mathscr {F})^\vee$, for every coherent sheaf $\mathscr {F}$ on *X*. In the particular, for $\mathscr {F}= \mathscr {O}_X$ one obtains an isomorphism $H^{-i}(\omega ^\bullet _X) \simeq H^i(X,\mathscr {O}_X)^\vee$. We consider the distinguished triangle:

$$\begin{aligned} \omega _X[d] \longrightarrow \omega ^\bullet _X \longrightarrow \mathscr {E}\xrightarrow {+1}. \end{aligned}$$

Taking cohomology, we obtain an exact sequence

$$\begin{aligned}&H^{-i-1}(X,\mathscr {E}) \longrightarrow H^{-i}(X,\omega _X[d]) \simeq H^{d-i}(X,\omega _X) \longrightarrow H^{-i}(X,\omega ^\bullet _X) \\&\quad \simeq H^i(X,\mathscr {O}_X)^\vee \longrightarrow H^{-i}(X,\mathscr {E}) \end{aligned}$$

However, noting that $\mathscr {H}^{-q}(\mathscr {E}) = \mathscr {H}^{-q}(\omega ^\bullet _X)$ for by $q<d$ and using [49, Tag 0ECM] along with the spectral sequence:

$$\begin{aligned} H^p(X,\mathscr {H}^q(\mathscr {E})) \Rightarrow H^{p+q}(X,\mathscr {E}), \end{aligned}$$

we see that $H^{-j}(X,\mathscr {E}) = 0$ for $0 \le j < k$. This finishes the proof. $\square$

We also recall that Serre properties behave well with respect to the procedure of taking geometric generic fibres.

#### Proposition 2.5

Let *X* be a variety over *k* and let *K*/*k* be a field extension. Then *X* satisfies property $S_k$ if and only if $X \otimes _k K$ does. In particular, if $f :X \rightarrow Y$ is a morphism of varieties over *k* and *X* satisfies property $S_k$, then the geometric generic fibre of *f* satisfies $S_k$ too.

#### Proof

For the first part of the proposition we refer to [20, Corollaire 6.7.8]. For the second, we just observe that $S_k$ is a local property and is therefore inherited by the generic fibre. Consequently, using the first part it also holds for the geometric generic fibre. $\square$

Concerning the relation between global *F*-splitting and ordinarity, we need the following result. We provide the proof for the reader’s convenience and because the classical reference does not treat the singular case. A slightly different argument also already appeared in [24, Section 2.4].

#### Proposition 2.6

[35, Proposition 9] Let *X* be a normal projective variety over *k* with $K_X \sim 0$. Then *X* is weakly ordinary if and only if it is globally *F*-split.

#### Proof

Let *U* be the regular locus of *X*, and set $d:= \dim X$. Then we have: 
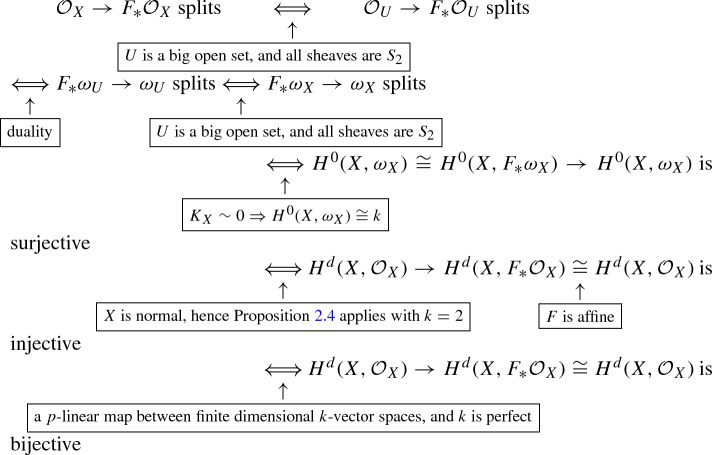


## Witt vector and *p*-adic cohomology

In this section we recall the basic properties of Witt vector and *p*-adic cohomology groups and direct images. For the results related to Witt vector cohomology we refer to [3] and [11] for original accounts, and to [18, Section 2.5] for an accessible summary. The necessary results concerning *p*-adic cohomology can be found in the classical reference [45, Exposé V, VI]. For the sake of clarity, we recall most of the definitions and some useful properties.

### Basics of $W\mathcal {O}$-cohomology

First, we recall that for every scheme defined over $\mathbb {F}_p$ there exists a sequence of schemes $W_n(X)$, for $n \ge 1$, underlying the same topological space *X* and with varying sheaves of rings $W_n\mathcal {O}_X$ given by Witt vectors of length *n*, see [44, II, Section 6] or [18, Section 2.5],. We define the sheaf $W\mathcal {O}_X$ on *X* as the following inverse limit taken in the category ${{\,\mathrm{Ab}\,}}(X)$ of sheaves of abelian groupsWe note that unlike $W_n(X) = (X,W_n\mathcal {O}_X)$ the pair $(X,W\mathcal {O}_X)$ is not a scheme.

As will be stated in Definition 3.8, one of the defining properties of $W\mathcal {O}$-rational singularities is that $R^i f_* W\mathcal {O}_{Y,\mathbb {Q}}=0$ for every integer $i>0$ and every quasi-resolution of the singularities (see Definition 3.6). The question is however how one defines $R^i f_* W\mathcal {O}_{Y,\mathbb {Q}}$. The naive idea is to let $R^i f_* W\mathcal {O}_{Y,\mathbb {Q}}$ be defined as $(R^i f_* W\mathcal {O}_{Y}) \otimes _{\mathbb {Z}} \mathbb {Q}$. According to the understanding of the authors, it is not known whether this definition is the correct one. The main reason is that it is not known whether the $p^{\infty }$-torsion in $R^i f_* W \mathcal {O}_{Y}$ is annihilated by $p^M$ for a single integer $M>0$ or not. If the inverse system $\{ R^i f_* W_n \mathcal {O}_X \}_{n \in \mathbb {N}}$ satisfied the Mittag–Leffler condition, then there would not be $p^\infty$-torsion of infinite order by the arguments of [3, Prop 2.10]. However, to the best of our knowledge, it is again not known whether this Mittag–Leffler property always holds true. Hence, to avoid these problems, one defines $R^i f_* W\mathcal {O}_{Y,\mathbb {Q}}$ in a way so that its $p^{\infty }$-torsion is annihilated by $p^M$ for a single integer $M>0$. In particular, the definition becomes slightly cumbersome, in a sense that it uses the notion of localization of a category in a Serre subcategory.

#### Remark 3.1

The notion of localization of a category in a full subcategory is well-explained in [49, Tag 02MN]. For the original account we refer to [16, Chapitre III].

In our case, this is applied to the full subcategory ${{\,\mathrm{Ab}\,}}(X)_{{{\,\mathrm{b-tors}\,}}}$ of the category ${{\,\mathrm{Ab}\,}}(X)$ of abelian sheaves on a scheme *X*, defined by the formula:

$$\begin{aligned} A \in \mathrm{Ob}\left( {{\,\mathrm{Ab}\,}}(X)_{{{\,\mathrm{b-tors}\,}}}\right) \iff \exists N \in \mathbb {Z}\setminus 0 \ : \ N \cdot {{\,\mathrm{{id}}\,}}_A = 0. \end{aligned}$$

The category obtained by localizing ${{\,\mathrm{Ab}\,}}(X)$ in ${{\,\mathrm{Ab}\,}}(X)_{{{\,\mathrm{b-tors}\,}}}$ is denoted ${{\,\mathrm{Ab}\,}}(X)_{\mathbb {Q}}$. The category ${{\,\mathrm{Ab}\,}}(X)_{\mathbb {Q}}$ is called the category of $\mathbb {Q}$-localized abelian sheaves, and it is an abelian category. One intuitive description of this localization is formally inverting all arrows in ${{\,\mathrm{Ab}\,}}(X)$ whose kernel and cokernel are in ${{\,\mathrm{Ab}\,}}(X)_{{{\,\mathrm{b-tors}\,}}}$. We denote the natural projection functor by $q :{{\,\mathrm{Ab}\,}}(X) \rightarrow {{\,\mathrm{Ab}\,}}(X)_{\mathbb {Q}}$ or simply $(-)_{\mathbb {Q}}$, and we note that:

- $q(A) = 0$ if and only if $A \in {{\,\mathrm{Ab}\,}}(X)_{{{\,\mathrm{b-tors}\,}}}$ [11, 3.7.2, line 6],
- ${{\,\mathrm{Hom}\,}}_{{{\,\mathrm{Ab}\,}}(X)_{\mathbb {Q}}} (q(\mathcal {F}) , q(\mathcal {G}))= {{\,\mathrm{Hom}\,}}_{{{\,\mathrm{Ab}\,}}(X)}(q(\mathcal {F}), q(\mathcal {G})) \otimes _{\mathbb {Z}} \mathbb {Q}$ for any $\mathcal {F}, \mathcal {G}\in {{\,\mathrm{Ab}\,}}(X)$ [11, Prop 3.7.4], and
- the functor *q* is exact [49, Tag 02MN] .

In particular, the above setting is used to define $W\mathcal {O}_{X,\mathbb {Q}}$:The next goal is to define the derived pushforwards of $W\mathcal {O}_{X,\mathbb {Q}}$. By [11, Cor 3.7.5], if $f :X \rightarrow Y$ is a morphism of varieties over *k*, the natural functor $f_* :{{\,\mathrm{Ab}\,}}(X) \rightarrow {{\,\mathrm{Ab}\,}}(Y)$ descends to localized categories yielding $f_* :{{\,\mathrm{Ab}\,}}(X)_{\mathbb {Q}} \rightarrow {{\,\mathrm{Ab}\,}}(Y)_{\mathbb {Q}}$. Moreover, by [11, Prop 3.7.7], both functors admit total right derived functors $Rf_*$ which satisfy compatibility condition expressed by commutativity of the diagram:As a consequence, the cohomology groups satisfy the equation $(R^if_*A)_{\mathbb {Q}} \simeq R^if_*(A_{\mathbb {Q}})$, and hence this setup allows ups to define $R f_* W \mathcal {O}_{X,\mathbb {Q}}$:

#### Definition 3.2

Let $f :X \rightarrow Y$ be a morphism of varieties over *k*. We define the *i*th derived pushforward of $W\mathcal {O}_{X,\mathbb {Q}}$ by $R^if_* \left( W\mathcal {O}_{X,\mathbb {Q}} \right) \in {{\,\mathrm{Ab}\,}}(Y)_{\mathbb {Q}}$. In particular, the Witt vector cohomology $H^i(X,W\mathcal {O}_{X,\mathbb {Q}}) \in {\mathrm{Mod}(W(k))}_{\mathbb {Q}}$ of a scheme *X* defined over a perfect field *k* is given by the formula:

$$\begin{aligned} H^i(X,W\mathcal {O}_{X,\mathbb {Q}}) = R^i\Gamma \left( W\mathcal {O}_{X,\mathbb {Q}} \right) , \end{aligned}$$

where $\Gamma :X \rightarrow {{\,\mathrm{Spec}\,}}(k)$ is the structure map.

#### Remark 3.3

According to [18, Remark 2.17.(5)], if *X* is proper over *k*, then $H^i(X,W\mathcal {O}_{X,\mathbb {Q}})=0$ if and only if $H^i(X,W\mathcal {O}_{X}) \otimes _{\mathbb {Z}} \mathbb {Q}=0$. As explained in the first paragraph of the section, it is not known if the corresponding statement for a proper birational morphism holds true.

Witt vector cohomology relates to finite length Witt vector cohomology via the following results. These results are deduced from Grothendieck spectral sequence for the composition of derived functors $R\lim \circ Rf_* = Rf_* \circ R\lim$ applied to the system $n \mapsto W_n\mathcal {O}_X$ which satisfies Mittag-Leffler condition.

#### Proposition 3.4

[18, Lem 2.18] Let $f :X \rightarrow Y$ be a morphism of varieties over *k*. Then, for every $i \ge 0$, there exists a short exact sequence:

$$\begin{aligned} 0 \longrightarrow R^1 \varprojlim _{n} \left( R^{i-1}f_*W_n\mathcal {O}_X \right) \longrightarrow R^if_* W\mathcal {O}_X \longrightarrow \left( \varprojlim _n R^if_* W_n\mathcal {O}_X \right) \longrightarrow 0. \end{aligned}$$

In particular, if *X* is a proper scheme over *k*, then for all *i* the system $n \mapsto H^i(X,W_n\mathcal {O}_X)$ satisfies the Mittag–Leffler condition, and hence

We will also use the following result relating Witt vector cohomology with the rigid cohomology, that is, the substitute of crystalline cohomology in the singular setting. Since the main application of the result is actually the comparison between Witt vector and *p*-adic cohomology (see Theorem 3.17 and Corollary 3.18), and rigid cohomology can be treated as a black box for this purpose, we do not include any details, referring to the standard literature on the topic such as [5]. We only recall that *K* denotes the fraction field of the ring of Witt vectors *W*(*k*) and that rigid cohomology admits a structure of an *F*-isocrystal over *k* (i.e., a *K*-vector space with a Frobenius linear endomorphism). By Dieudonné–Manin decomposition all such objects admit slope decomposition. For an *F*-isocrystal *M*, by $M^{<c}$ we denote the part of *M* where Frobenius acts with slopes $<c$.

#### Theorem 3.5

[3, Theorem 1.1] Let *k* be a perfect field of characteristic $p > 0$ and let *X* be a *k*-scheme of finite type. There exists a functorial isomorphism:where $K:={{\,\mathrm{Frac}\,}}(W(k))$.

### $W\mathcal {O}$-singularities

Since singular schemes naturally show up in our approach to the main theorem, and since we use a Witt-vector cohomology based invariant to show non-uniruledness (see Proposition 4.7), we need a characteristic $p>0$ notion of singularities allowing for the control of Witt vector cohomology. We will use the notion of $W\mathcal {O}$-rational singularities introduced in the papers [4, 11].

The definition uses the notion of a *quasi-resolution* (see [11, Definition 4.3.1]). For the sake of clarity we recall some of its details. We say that a normal equidimensional scheme *X* defined over a field *k* is a *finite quotient* if there exists a finite surjective morphism $Y \rightarrow X$ from a scheme *Y* smooth over *k*. A scheme is a *topological finite quotient* if there exists a universal homeomorphism from (equiv. to) a finite quotient as defined above.

#### Definition 3.6

[11, Definition 4.3.1] We say that a morphism between two integral varieties $f :X \rightarrow Y$ is a *quasi-resolution* if the following conditions are satisfied:

1. *X* is a topological finite quotient,
2. *f* is projective, surjective, and generically finite,
3. the extension of the function fields $k(Y) \subset k(X)$ is purely inseparable.

We note that if *Y* is normal the last condition is actually equivalent to the Stein factorization $X' \rightarrow Y$ being a universal homeomorphism. By the work of de Jong [13] for every variety *X* over a perfect field *k* there exists a quasi-resolution with *X* as a target (see [11, Remark 4.3.2]). More precisely, the alterations of de Jong could be chosen to be compositions of quotients of smooth varieties by finite group actions and quasi-resolutions.

#### Lemma 3.7

An étale base change of a quasi-resolution is a quasi-resolution.

#### Proof

Let $X \rightarrow Y$ be a quasi-resolution and let $Y' \rightarrow Y$ be an étale morphism. Take $U \rightarrow X$ to be a morphism furnishing the topological finite quotient structure on *X*. We consider the following diagram of cartesian squares:The morphism $U' \rightarrow U$ is étale and therefore $U'$ is smooth. Consequently $U' \rightarrow X'$ yields a topological finite quotient structure on $X'$. Using standard base change properties the map $X' \rightarrow Y'$ satisfies the conditions from Definition 3.6, and hence $X' \rightarrow Y'$ is a quasi-resolution as desired. $\square$

#### Definition 3.8

[11, Definition 4.4.1 and Proposition 4.4.6] Let *X* be a variety over *k*. We say that *X* has $W\mathcal {O}$-*rational singularities* if and only if for every (equiv. some) quasi-resolution $f :Y \rightarrow X$ the following two conditions are satisfied:

1. the natural map induces an isomorphism $W\mathcal {O}_{X,\mathbb {Q}} \simeq f_*W\mathcal {O}_{Y,\mathbb {Q}}$,
2. $$R^if_*W\mathcal {O}_{Y,\mathbb {Q}} = 0$$, for every $$i > 0$$.

#### Remark 3.9

We note that by compatibility of derived functor of $f_*$ and localization the following statements concerning the definition hold true:

- if *X* is normal then the first condition is automatically satisfied,
- the conditions are equivalent to the fact that the natural map in the derived category of $\mathbb {Q}$-localized abelian sheaves $W\mathcal {O}_{X,\mathbb {Q}} \rightarrow Rf_*W\mathcal {O}_{Y,\mathbb {Q}}$ is an isomorphism.

In what follows we say that a morphism satisfying the above conditions is $W\mathcal {O}$-*rational*. Using this notion we may rephrase $W\mathcal {O}$-rationality of a scheme *X* by postulating the existence of a $W\mathcal {O}$-rational quasi-resolution $Y \rightarrow X$.

We thank Kay Rülling for the following result and the subsequent proof of étale invariance of $W\mathcal {O}$-rational singularities.

#### Proposition 3.10

Let $f :Y \rightarrow X$ be a proper, surjective and generically finite morphism of varieties over *k*. Then the following conditions are equivalent:

1. the morphism *f* is $W\mathcal {O}$-rational, that is, $W\mathcal {O}_{X,\mathbb {Q}} \rightarrow f_*W\mathcal {O}_{Y,\mathbb {Q}}$ is an isomorphism and $R^if_*W\mathcal {O}_{Y,\mathbb {Q}} = 0$, for every $i>0$,
2. there exists an integer $M>0$ such that for every $n \ge 1$ the abelian sheaves $f_*W_n\mathcal {O}_Y/W_n\mathcal {O}_X$ and $R^if_*W_n\mathcal {O}_X$, for $i \ge 1$, are annihilated by $p^M$.

#### Proof

We first prove that (2) $\Rightarrow$ (1). We set $C_n = f_*W_n\mathcal {O}_Y/W_n\mathcal {O}_X$ and consider the following exact sequence of systems of abelian sheaves on *X*:

$$\begin{aligned} 0 \longrightarrow \{ W_n\mathcal {O}_X \}_{n \in \mathbb {N}} \longrightarrow \{ f_*W_n\mathcal {O}_Y \}_{n \in \mathbb {N}} \longrightarrow \{ C_n \}_{n \in \mathbb {N}} \longrightarrow 0. \end{aligned}$$

Since for every scheme *V* the sheaf $W_n\mathscr {O}_V$ is set-theoretically just a sheaf of tuples of elements of $\mathscr {O}_V$, the restriction maps $W_n\mathscr {O}_V(U) \rightarrow W_m\mathscr {O}_V(U)$, for $n \ge m$ and $U \subset V$ an every open subset, are surjective and hence both systems $n \mapsto W_n\mathscr {O}_X$ and $n \mapsto f_*W_n\mathscr {O}_Y$ satisfy the Mittag-Leffler condition. We recall that sections of $f_*W_n\mathscr {O}_Y$ are actually given by sections of $W_n\mathscr {O}_Y$ on appropriate open subsets so the above argument is sufficient for both systems. Consequently, using the long exact sequence of $R^\bullet \varprojlim$ groups, we obtain:
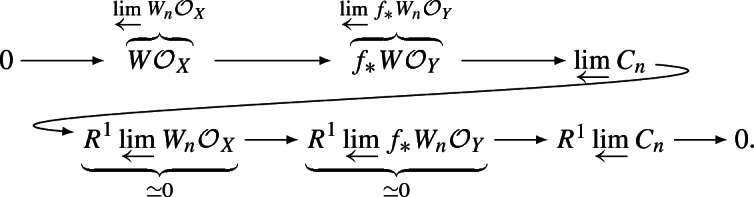


Taking images in the localized category, and hence annihilating the elements $\varprojlim C_n$ and $R^1\varprojlim C_n$, which are $p^M$-torsion, we obtain the desired statement for $i = 0$. Then using Proposition 3.4, we have an exact sequence

$$\begin{aligned} 0 \longrightarrow R^1\varprojlim R^{i-1}f_*W_n\mathcal {O}_Y \longrightarrow R^if_*W\mathcal {O}_Y \longrightarrow \varprojlim R^if_*W_n\mathcal {O}_Y \longrightarrow 0. \end{aligned}$$

Again all the elements in the relevant inverse systems are annihilated by $p^M$, for some fixed *M*, and therefore both the right and left elements in the above exact sequence are annihilated by $p^M$ as well. Consequently, the middle term is $p^{2M}$-torsion and hence the implication is proven.

We now approach (1) $\Rightarrow$ (2). For this purpose we apply Remark 3.9 and the definition of ${{\,\mathrm{Ab}\,}}(X)_{\mathbb {Q}}$ to see that there is an integer $N>0$ such that $\mathcal {Q}_i$ is $p^N$-torsion, where3.10.aConsider the exact sequence3.10.bThe long exact sequence obtained from (3.10.b) by applying derived pushforwards sandwiches $R^i f_* W_n \mathcal {O}_Y$ between $\mathcal {Q}_i$ and $\mathcal {Q}_{i+1}$. As the latter are $p^N$-torsion, we obtain that $R^i f_* W_n \mathcal {O}_Y$ is $p^{2N}$ torsion for all $i \ge 0$ and all $n \ge 1$.

We have to show the same statement for $\mathcal {Q}_0$ too. For that, consider the following diagram where all rows and columns are exact. To see this, start with the two leftmost non-zero columns that we already know are exact. Apply then the snake lemma first two the first three non-zero entries of these columns, and then to the second and third non-zero rows.As, we already know that $\mathcal {E}$ and $\mathcal {Q}_0$ are $p^N$ torsion, we obtain that $C_n$ is indeed $p^{2N}$ torsion for every integer $n \ge 1$. That is, we obtain the statement of (2), for $M = 2N$. $\square$

As a corollary of Proposition 3.10 and [11, Proposition 4.4.9], we obtain:

#### Proposition 3.11

Let *X* be a variety over *k*. Then the following assertions hold:

1. the notion of $W\mathcal {O}$-rationality is local in the étale topology,
2. if $X' \rightarrow X$ is a universal homeomorphism then *X* is $W\mathcal {O}$-rational if and only if $X'$ is.

#### Proof

The result (2) is proven [11, Proposition 4.4.9]. For (1), we observe that [23, I, Proposition 1.5.8] implies that for every surjective étale morphism $U \rightarrow X$ the associated map $W_n(U) \rightarrow W_n(X)$ is also surjective étale, and consequently for a quasi-resolution $f :Y \rightarrow X$ we obtain a diagram with surjective étale rows:and $f_U :Y \times _X U \rightarrow U$ is a quasi-resolution according to Lemma 3.7. We claim that the diagram is in fact cartesian. For this purpose we observe that there exists a natural morphism $W_n(Y \times _X U) \rightarrow W_n(Y) \times _{W_n(X)} W_n U$ which is étale, we use [23, I,Proposition 1.5.8] for $Y \times _X U \rightarrow Y$ and the cancellation property for étale maps. The morphism becomes an identity when restricted via the closed immersion $Y \times _X U \rightarrow W_n(Y) \times _{W_n(X)} W_n U$ and hence is an identity itself by invariance of the étale site under nilpotent thickenings (see [49, Tag 04DZ]).

Consequently, using flat base change (note that as above $W_n(\pi )$ is étale and hence flat), we see that

$$\begin{aligned} R^if_{U,*} W_n\mathcal {O}_{Y \times _X U} = W_n(\pi )^* R^if_* W_n\mathcal {O}_Y. \end{aligned}$$

Since $W_n(\pi )$ is in fact faithfully flat, this implies that for every $M>0$ the integer $p^M$ annihilates $R^if_* W_n\mathcal {O}_Y$ if and only if it annihilates $R^if_{U,*} W_n\mathcal {O}_{Y \times _X U}$, which finishes the proof by Proposition 3.10. $\square$

#### Proposition 3.12

If *X* is a $W\mathcal {O}$-rational variety over *k*, and *K*/*k* is a separable (possibly infinite) algebraic field extenstion, then the variety $X_K$ is $W\mathcal {O}$-rational.

#### Proof

Let $f :Y \rightarrow X$ be a $W\mathcal {O}$-rational quasi-resolution, and let $\{K_\lambda \}_{\lambda \in I}$ be an ascending system of finite separable extension $k \subset K_\lambda \subset K$ such that $\bigcup _\lambda K_\lambda = K$. Set $\pi :X_K \rightarrow X$, $\psi :Y_K \rightarrow Y$, $\pi _{K_\lambda } :X_{K_\lambda } \rightarrow X$ and $\psi _{K_\lambda } :Y_{K_\lambda } \rightarrow Y$ to be the natural maps. We shall prove that the base change $f_K :Y_K \rightarrow X_K$ is a $W\mathcal {O}$-rational quasi-resolution. It is a quasi-resolution by the arguments of Lemma 3.7. In order to see that it is also $W\mathcal {O}$-rational we reason as follows. Using Proposition 3.10, we choose an *M* such that $p^M \cdot R^if_*W_n\mathcal {O}_Y = 0$. We claim it is also true for $f_K$ which concludes the proof using Proposition 3.10 once again. We consider the following diagrams, where by abuse of notation we identify every morphism *g* with $W_n(g)$:Since the map $\pi _K$ is affine, we see that $p^M \cdot R^i f_{K,*} W_n\mathcal {O}_{X_K} = 0$ if and only if $p^M \cdot \pi _{K,*} R^i f_{K,*} W_n\mathcal {O}_{X_K} = 0$. By the commutativity of the diagram above and the fact that $\psi _K$ is also affine, this is equivalent to the vanishing $p^M \cdot R^i f_*(\psi _{K,*} W_n \mathcal {O}_{Y_K})$. Since the forgetful functor from sheaves of rings to sheaves of sets preserves direct limits and $W_n$ is the *n*-fold self-product set theoretically, the pushforward $\psi _{K,*} W_n \mathcal {O}_{Y_K}$ is in fact isomorphic to

$$\begin{aligned} \varinjlim _{\lambda } \psi _{K_\lambda ,*}W_n \mathcal {O}_{Y_{K_\lambda }}. \end{aligned}$$

Now, since direct images commute with direct limits for quasi-compact quasi-separated morphisms (see [49, Tag 07U6]) we see that

$$\begin{aligned} p^M \cdot R^i f_*(\psi _{K,*} W_n \mathcal {O}_{Y_K})&\simeq p^M \cdot R^i f_*(\varinjlim _{\lambda } \psi _{K_\lambda ,*} W_n \mathcal {O}_{Y_{K_\lambda }}) \\&\simeq p^M \cdot \varinjlim _{\lambda } \pi _{K_\lambda ,*} R^i f_{K_\lambda ,*} W_n\mathcal {O}_{Y_{K_\lambda }} \\&= \varinjlim _{\lambda } p^M \cdot \pi _{K_\lambda ,*} R^i f_{K_\lambda ,*} W_n\mathcal {O}_{Y_{K_\lambda }}. \end{aligned}$$

However, since the morphisms $X_{K_\lambda } \rightarrow X$ are in fact étale, by the argument in Proposition 3.11 all the terms in the last direct limit vanish which finishes the proof. $\square$

#### Remark 3.13

We note that one would hope that a similar proof actually works for an arbitrary extension. Unfortunately, a purely inseparable extension of height *k* leads to the substitution of the exponent *M* by $M+k$. The limiting procedure cannot be therefore performed.

### Primer on *p*-adic cohomology

For the sake of completeness, in this section we present the basic results concerning *p*-adic cohomology and direct images which are going to be necessary in our argument. Most the results are contained in [45, Exposé V “Systemes projectifs *J*-adiques”] and [45, Exposé VI “Cohomologie $\ell$-adique”] by Jean-Pierre Jouanolou where the formalism of $\ell$-adic sheaves is developed. We emphasize that the standard assumption $\ell \ne p$ showing up in $\ell$-adic cohomology considerations is not necessary for neither of the results in *loc. cit*.

We begin with a short description of the necessary notion of Artin-Rees category ${{\,\mathrm{AR}\,}}(\mathbb {Z}_p)$ of *p*-adic sheaves. We refer to [45, Exposé V, §2.2] for the original account. All the sheaves considered here are in étale topology. The objects of the category are projective systems

$$\begin{aligned} \mathscr {F}_\bullet = \left( \ \mathscr {F}_n \ ,\ \varphi ^\mathscr {F}_n :\mathscr {F}_n \rightarrow \mathscr {F}_{n-1}\ \right) _{n \in \mathbb {Z}} \end{aligned}$$

of constructible (and hence torsion) sheaves of $\mathbb {Z}_p$-modules such that $\mathscr {F}_n = 0$, for $n \ll 0$. The morphisms are defined in a slightly subtle way, by the formula:

$$\begin{aligned} {{\,\mathrm{Hom}\,}}_{{{\,\mathrm{AR}\,}}(\mathbb {Z}_p)}(\mathscr {F}_\bullet ,\mathscr {G}_\bullet ) = \varinjlim _{d \in \mathbb {Z}} \ {{\,\mathrm{Hom}\,}}_\mathrm{systems}(\mathscr {F}_\bullet [d],\mathscr {G}_\bullet ) \end{aligned}$$

where $\mathscr {F}[d]$ denotes the system defined by the association $\mathscr {F}_\bullet [d]_n = \mathscr {F}_{n+d}$, and the colimit is taken along the maps ${{\,\mathrm{Hom}\,}}_\mathrm{systems}(\mathscr {F}_\bullet [d],\mathscr {G}_\bullet ) \rightarrow {{\,\mathrm{Hom}\,}}_\mathrm{systems}(\mathscr {F}_\bullet [d+1],\mathscr {G}_\bullet )$ induced by the diagram:The right square in the diagram is in fact a morphism $\mathscr {F}[d+1] \rightarrow \mathscr {F}[d]$ of systems, and the above map is a composition therewith.

We remark that the above definition is in fact equivalent to the localization of the category of systems of sheaves of $\mathbb {Z}_p$-modules satisfying the condition $\mathscr {F}_n = 0$, for $n \ll 0$, by the subcategory of *null systems*, that is, systems $\mathscr {N}_{\bullet }$ for which the natural map $\mathscr {N}[d] \rightarrow \mathscr {N}$, induced by composition of *d* transition maps, is zero for some $d \in \mathbb {N}$ (see [45, Exposé V, Proposition 2.4.3]). A system $\mathscr {F}_\bullet = (\mathscr {F}_n)$ is *strict* if $\mathscr {F}_n = 0$, for $n<0$, and for $n \ge 0$ the sheaf $\mathscr {F}_n$ is annihilated by $p^{n+1}$ and satisfies $\mathscr {F}_n \otimes _{\mathbb {Z}/p^{n+1}} \mathbb {Z}/p^n \simeq \mathscr {F}_{n-1}$.

#### Definition 3.14

(*p*-adic sheaves) We define the categories of $\mathbb {Z}_p$ and $\mathbb {Q}_p$ sheaves as follows:

1. the category of constructible $\mathbb {Z}_p$-sheaves is defined as the full subcategory of the category ${{\,\mathrm{AR}\,}}(\mathbb {Z}_p)$ of systems isomorphic to a strict constructible system.
2. the category of constructible $\mathbb {Q}_p$-*sheaves* (see [45, Exposé VI, Définition 1.4.3]) is defined as the localization of the category of $\mathbb {Z}_p$-sheaves by the Serre subcategory of sheaves annihilated by a fixed power of *p* (see Remark 3.1 for a brief description of the localization procedure).

Let $f :X \rightarrow Y$ be a proper morphism of locally noetherian schemes. The most important reason for the introduction of the above admittedly complex definition is that it allows for the direct images of a $\mathbb {Z}_p$-sheaf $\mathscr {F}= (\mathscr {F}_n)_{n \in \mathbb {N}}$ to be well-defined using the formula:

$$\begin{aligned} R^if_*\mathscr {F}= (R^if_*\mathscr {F}_n)_{n \in \mathbb {N}}. \end{aligned}$$

We remark that the above higher direct image systems are often not strict constructible themselves. However, by the proper base change for $\mathbb {Z}/p^n$-constructible sheaves and the main result of [45, Exposé VI, §2.2], they are Artin–Rees isomorphic to a strict constructible system. In fact, the only condition in Definition 3.14 which is likely not satisfied by the system $(R^if_*\mathscr {F}_n)_{n \in \mathbb {N}}$ is strictness, which however holds after an Artin–Rees isomorphism. Moreover, if $f :\overline{X} \rightarrow {{\,\mathrm{Spec}\,}}(\overline{k})$ is a structure morphism of a base change over the algebraic closure of a variety *X*/*k*, we recover the usual definition of *p*-adic cohomology using [45, Exposé VI, 1.2 “$\mathbb {Z}_\ell$ faisceaux constants tordus constructible”]. We note that in this case all sheaves $R^if_*\mathbb {Z}/p^n$ are in fact torsion constant and hence clearly satisfy requirements of loc. cit.

We shall need the following standard results concerning behaviour of constructible $\mathbb {Z}_p$-sheaves. We remark that although the following results are stated in [45, Exposé VI] only for $\mathbb {Z}_p$-sheaves, the author explains in [45, Exposé VI, 2.2.5] that all the results can be carried through to the $\mathbb {Q}_p$ setting using straightforward localization.

#### Proposition 3.15

[45, Exposé VI, Proposition 2.2.4] Let $f :X \rightarrow Y$ and $g :Y \rightarrow Z$ be proper finite type morphisms of locally noetherian schemes. Then for every constructible *p*-adic sheaf $\mathscr {F}$ there exists a convergent Leray spectral sequence:

$$\begin{aligned} E^{pq}_2 = R^pg_*(R^qf_*\mathscr {F})\Rightarrow R^{p+q}(g \circ f)_* \mathscr {F}. \end{aligned}$$

In particular, if *Y* is a proper variety defined over an algebraically closed field and $f :X \rightarrow Y$ is proper finite type morphism then there exists a convergent Leray spectral sequence:

$$\begin{aligned} E^{pq}_2 = H^p_{{\mathrm{K}\acute{\mathrm{e}}\mathrm{t}}}(Y,R^qf_*\mathbb {Q}_p) \Rightarrow H^{p+q}_{{\mathrm{K}\acute{\mathrm{e}}\mathrm{t}}}(Y,\mathbb {Q}_p). \end{aligned}$$

In fact, the result in *loc. cit* works for higher direct images with compact support but we will only need it in the stated generality.

#### Proposition 3.16

[45, Exposé VI, 2.2.3 B)] Suppose that the diagramis a cartesian square of locally noetherian schemes. Assume that the morphism *f* is proper. Then for every constructible $\mathbb {Z}_p$-sheaf $\mathscr {F}$ (resp. $\mathbb {Q}_p$-sheaf) the natural base change map:

$$\begin{aligned} u^*R^if_*\mathscr {F}\rightarrow R^ig_*(v^*\mathscr {F}) \end{aligned}$$

is an isomorphism.

#### Theorem 3.17

[10, Thèoréme 5.1] For every proper scheme *X* defined over an algebraically closed field the following identification holds true:

$$\begin{aligned} H^\bullet _{{\mathrm{K}\acute{\mathrm{e}}\mathrm{t}}}(X,\mathbb {Q}_p) \otimes K \simeq H^\bullet _\mathrm{rig}(X)^{=0}, \end{aligned}$$

where $$K:={{\,\mathrm{Frac}\,}}(W(k))$$.

As a direct combination of Theorems 3.5 and 3.17 we obtain the following:

#### Corollary 3.18

For every finite type scheme *X* defined over an algebraically closed field the following identification holds true:

$$\begin{aligned} H^\bullet _{{\mathrm{K}\acute{\mathrm{e}}\mathrm{t}}}(X,\mathbb {Q}_p) \otimes K \simeq H^\bullet (X,W\mathcal {O}_{X,\mathbb {Q}})^{=0}, \end{aligned}$$

where $$K:={{\,\mathrm{Frac}\,}}(W(k))$$.

### *p*-adic cohomology and $W\mathcal {O}$-rational morphisms

In Corollary 3.18 we saw that the absolute *p*-adic cohomology is closely related to the Witt vector cohomology. The subsequent lemma provides a similar relation in the relative setting. We only present this simplistic version of the theorem, because a fully-fledged comparison will most likely require detailed application of rigid cohomology and *F*-isocrystals.

The main tool that we utilize is the system of Artin–Schreier–Witt sequences exact in étale topology:

3.18.a $$\begin{aligned} 0 \longrightarrow \mathbb {Z}/p^n \longrightarrow W_n\mathcal {O}_X \xrightarrow {1 - F} W_n\mathcal {O}_X \longrightarrow 0. \end{aligned}$$

#### Lemma 3.19

Let $f :X \rightarrow Y$ be a proper $W\mathcal {O}$-rational morphism (see Remark 3.9). Then $f_*\mathbb {Q}_p = \mathbb {Q}_p$ and $R^if_*\mathbb {Q}_p = 0$ for every $i>0$, or equivalently $\mathbb {Q}_p \simeq Rf_*\mathbb {Q}_p$ via the natural map.

#### Proof

Looking at degree $i>1$ terms of the long exact sequences of cohomology associated to (3.18.a) we obtain exact sequences:

3.19.b $$\begin{aligned} \cdots \longrightarrow R^{i-1}f_*W_n\mathcal {O}_X \longrightarrow R^if_*\mathbb {Z}/p^n \longrightarrow R^if_*W_n\mathcal {O}_X \longrightarrow \cdots . \end{aligned}$$

Since *f* is a $W\mathcal {O}$-rational, according to Proposition 3.10, the left and right terms of (3.19.b) are annihilated by a fixed number $p^M$ and hence for every $n>0$ the middle term is killed by $p^{2M}$. Using Definition 3.14, this directly implies that $R^if_*\mathbb {Q}_p = 0$, for $i>1$. For low degrees, for every $n>0$ we consider the diagram of sheaves on the étale site of *Y*: A priori, we only know that the leftmost three non-zero columns, as well as the middle row and the row above that are exact in (3.19.c). Then, Snake lemma applied to the first three non-zero entries of these two rows yields, that the row below the middle row is also exact. Finally, Snake lemma applied to the second and the third non-zero column yields that also the rightmost column is exact. So, eventually we obtain that all columns and rows of (3.19.c) are exact.

By the middle columns of (3.19.c) we obtain that $C_n$ are annihilated by $p^{M}$ for every $n>0$. Then, the lowest non-zero row of (3.19.c) implies that $K_n$ are annihilated by $p^{2M}$ for every $n>0$. Again, using Definition 3.14 and the fact that localization by Serre subcategories is an exact functor, this implies that $f_* \mathbb {Q}_p = \mathbb {Q}_p$. Moreover, for $i=1$, we observe that (3.19.b) and the middle row of (3.19.c) lead to the exact sequence

$$\begin{aligned} 0 \longrightarrow {{\,\mathrm{{coker}}\,}}\left( f_*(1 - F_X)\right) \longrightarrow R^1f_*(\mathbb {Z}/p^n)_X \longrightarrow R^1f_*W_n\mathcal {O}_X. \end{aligned}$$

Since both ${{\,\mathrm{{coker}}\,}}\left( f_*(1 - F_X)\right)$, which injects into ${{\,\mathrm{{coker}}\,}}\left( C_n \rightarrow C_n\right)$, and $R^1f_*W_n\mathcal {O}_X$ are $p^{M}$-torsion, the sheaf $R^1f_*(\mathbb {Z}/p^n)_X$ is $p^{2M}$-torsion for every $n>0$ and hence $R^1f_*\mathbb {Q}_p = 0$. This finishes our proof. The equivalence with the fact that the natural map $\mathbb {Q}_p \rightarrow Rf_*\mathbb {Q}_p$ is a quasi-isomorphism is now clear. $\square$

In the spirit of Remark 3.9, the morphism satisfying the properties in stated in Lemma 3.19 is called $\mathbb {Q}_p$-*rational*. That is a proper $f :X \rightarrow Y$ is $\mathbb {Q}_p$-rational if $f_*\mathbb {Q}_p = \mathbb {Q}_p$ and $R^if_*\mathbb {Q}_p = 0$ for every $i>0$, or equivalently $Rf_*\mathbb {Q}_p = \mathbb {Q}_p$. It turns out that we can in fact define a well-behaved family of $\mathbb {Q}_p$-rational singularities. More precisely, we have the following:

#### Proposition 3.20

Let *X* be a variety defined over *k*. Let $f :Y \rightarrow X$ and $g :Z \rightarrow X$ be two quasi-resolutions. Then *f* is $\mathbb {Q}_p$-rational if and only if *g* is $\mathbb {Q}_p$-rational.

#### Proof

Let *U* be the quasi-resolution of $Y \times _X Z$ and let $\overline{g} :U \rightarrow Y$ and $\overline{f} :U \rightarrow Z$ be the maps induces by the projections, both $W\mathscr {O}$-rational and hence $\mathbb {Q}_p$-rational because *Y* and *Z* are $W\mathscr {O}$-rational. Setting $g \circ \overline{f} = h = f \circ \overline{g}$, we have the following series of isomorphisms:which yields the claim of the proposition. $\square$

#### Definition 3.21

($\mathbb {Q}_p$-rational singularities) Let *X* be a variety defined over *k*. We say that *X* is $\mathbb {Q}_p$-rational (has $\mathbb {Q}_p$-rational singularities) if there exists a $\mathbb {Q}_p$-rational quasi-resolution of singularities (equiv. every quasi-resolution is $\mathbb {Q}_p$-rational).

#### Proposition 3.22

Let $f :X \rightarrow T$ be a flat morphism of varieties defined over *k*, and let ${\overline{\eta }}$ be a geometric generic point of *T*. Assume that *X* is $W\mathcal {O}$-rational. Then the geometric generic fibre $X_{{\overline{\eta }}}$ is $\mathbb {Q}_p$-rational.

#### Proof

By restricting to an open subset we may assume that *T* is smooth. Let $\xi :Y \rightarrow X_{{\overline{\eta }}}$ be a quasi-resolution. As *Y* and $\xi$ are of finite type over $k\left( {\overline{\eta }} \right)$, both are defined over a finite extension *L* of $k(\eta )$. We now consider the normalization *U* of *T* in *L*. The decomposition of the extension $L/k(\eta )$ into a separable and purely inseparable part leads to a factorization of $U \rightarrow T$ into a separable morphism $V \rightarrow T$ and a universal homeomorphism $U \rightarrow V$ (normalizations in purely inseparable extensions are universal homeomorphisms by [49, Tag 0BRA]). Restricting to open subsets we may further assume that $V \rightarrow T$ is étale. To sum up, until now we manage to exhibit existence of:

- a morphism $U \rightarrow T$, which is a composition of an étale map and a universal homeomorphism, and
- a quasi-resolution $\pi :Z \rightarrow X_U$ such that $\xi = \pi _{{\overline{\eta }}}$.

Using Proposition 3.11, the scheme $X_U$ is $W\mathcal {O}$-rational and hence $\pi$ is a $W\mathcal {O}$-rational morphism. By Lemma 3.19, the morphism $\pi$ is also $\mathbb {Q}_p$-rational. Applying the proper base change (see Proposition 3.16) this property is inherited by $\xi = \pi _{{\overline{\eta }}}$ which finishes the proof. $\square$

#### Remark 3.23

Although Proposition 3.22 is sufficient for our purpose, it is admittedly a workaround. A clearer claim would be that the geometric generic fibre is in fact $W\mathcal {O}$-rational itself. We were unfortunately unable to prove it. The main obstacle is the lack of a proper base change type theorem, at the geometric generic point, for Witt vector higher direct images.

## Top Witt vector and *p*-adic cohomology

In this part of the paper we present a few results concerning top Witt vector cohomology of varieties. We begin with the following simple technical result which allows us to describe the top Witt vector cohomology in terms of coherent cohomology and relate it to the *p*-adic cohomology under arithmetic assumption of ordinarity. This result is most likely standard but we could not find a precise reference.

### Proposition 4.1

Let *X* be a proper variety of dimension $d = \dim X$ defined over a perfect field *k* of characteristic $p>0$. Assume that *X* is weakly ordinary, that is, the natural Frobenius action on $H^d(X,\mathcal {O}_X)$ is bijective, and that $H^{d-1}(X,\mathcal {O}_X) = 0$. Then $H^d(X,W\mathcal {O}_{X,\mathbb {Q}})$ is a *K*-vector space of dimension $g = \dim _k H^d(X,\mathcal {O}_X)$, andwhere $K:= {{\,\mathrm{Frac}\,}}W(k)$.

### Proof

First, we observe that by a straightforward inductive argument $H^{d-1}(X,W_n\mathcal {O}_X) = 0$, for every $n \ge 1$. Then, we claim that the map $F :H^d(X,W_n\mathcal {O}_X) \rightarrow H^d(X,W_n\mathcal {O}_X)$ induced by the Witt vector Frobenius is an isomorphism. This follows directly by induction on *n* from the five lemma applied to following diagram, whose rows are exact by the vanishing of finite length Witt vector cohomology in degree $d-1$.Taking limit with respect to *n* this implies that the Frobenius endomorphism of $H^d(X,W\mathcal {O}_X)$ is also an isomorphism. Using the vanishing of $H^{d-1}(X,\mathcal {O}_X)$ again, we see that connecting homomorphism $H^{d-1}(X,\mathcal {O}_X) \rightarrow H^d(X,W\mathcal {O}_X)$ in the long exact sequence associated with

$$\begin{aligned} 0 \longrightarrow W\mathcal {O}_X \xrightarrow {V} W\mathcal {O}_X \longrightarrow \mathcal {O}_X \longrightarrow 0. \end{aligned}$$

is zero. Therefore the map $V :H^d(X,W\mathcal {O}_X) \rightarrow H^d(X,W\mathcal {O}_X)$ is injective and hence $p = FV$ is injective as well. By Proposition 3.4, the natural map $H^d(X,W\mathcal {O}_X) \rightarrow \varprojlim _{n} H^d(X,W_n\mathcal {O}_X)$ is an isomorphism, which implies that $\bigcap _n V^n H^d(X,W\mathcal {O}_X) = \{0\}$ and therefore $H^d(X,W\mathcal {O}_X)$ is *V*-adically and hence *p*-adically separated. Consequently, using Nakayama’s lemma for the complete ring *W*(*k*) (see [34, Theorem 8.4]), we see that the torsion-free and *p*-adically separated module $H^d(X,W\mathcal {O}_X)$ is in fact free of rank:

$$\begin{aligned}&\dim _k H^d(X,W\mathcal {O}_X)/pH^d(X,W\mathcal {O}_X) = \dim _k H^d(X,W\mathcal {O}_X)/VH^d(X,W\mathcal {O}_X)\\&\quad = \dim _k H^d(X,\mathcal {O}_X), \end{aligned}$$

as desired. After localization, this yields the result concerning Witt vector cohomology.

For the claim related to *p*-adic cohomology, we take a limit of the cohomology exact sequences associated to (3.18.a) in order to obtain a short exact sequence (note that we use the vanishing in degree $d-1$):

$$\begin{aligned} 0 \longrightarrow H^d_{{\mathrm{K}\acute{\mathrm{e}}\mathrm{t}}}(X,\mathbb {Z}_p) \longrightarrow H^d(X,W\mathcal {O}_X) \xrightarrow {1 - F} H^d(X,W\mathcal {O}_X) \longrightarrow 0, \end{aligned}$$

which means that $H^d_{{\mathrm{K}\acute{\mathrm{e}}\mathrm{t}}}(X,\mathbb {Z}_p)$ are in fact Frobenius stable elements in the *F*-crystal $H^d(X,W\mathcal {O}_X)$. Since, as proven above, the morphism *F* acting on $H^d(X,W\mathcal {O}_X)$ is a *p*-linear automorphism, we may now use the standard result on *F*-crystals (see [25, 2.1.2]) which implies that $H^d(X,W\mathcal {O}_X)$ is generated by Frobenius stable elements. This yields the claim after localization. $\square$

### Remark 4.2

We remark that by the same argument using Proposition 3.4 we get that for every variety *X* of dimension $d = \dim X$ satisfying $H^d(X,\mathcal {O}_X) = 0$ the vanishing $H^d(X,W\mathcal {O}_{X,\mathbb {Q}}) = 0$ also holds.

We now proceed to the proof that both Witt vector and *p*-adic cohomology vanishes for uniruled $W\mathcal {O}$-rational varieties. We precede the actual statement with a few lemmata. Before stating them we recall that: if *X* and *Y* are integral, and *Y* is normal, then $u :X \rightarrow Y$ is a universal homeomorphism if and only if it is finite, surjective and purely inseparable (see [19, Prop. (3.5.8)]).

### Lemma 4.3

[11, Lemma 4.1.6 & Lemma 4.2.4] Let $f :Y \rightarrow X$ be a morphism of normal varieties over *k*. Then the following statements hold true for every $i>0$:

- if *f* is finite surjective then $f^* :H^i(X,W\mathcal {O}_{X,\mathbb {Q}}) \rightarrow H^i(Y,W\mathcal {O}_{Y,\mathbb {Q}})$ is injective,
- if *f* is a universal homeomorphism then $f^* :H^i(X,W\mathcal {O}_{X,\mathbb {Q}}) \rightarrow H^i(Y,W\mathcal {O}_{Y,\mathbb {Q}})$ is an isomorphism.

### Lemma 4.4

Let $f :X \rightarrow Y$ be a birational morphism of varieties of dimension *d*. Then for every $i>0$ we have

$$\begin{aligned} \dim \mathrm{Supp}(R^if_*W\mathcal {O}_{X,\mathbb {Q}}) < d-i. \end{aligned}$$

### Proof

We first recall the standard proof that $\dim \mathrm{Supp}(R^if_*\mathcal {O}_X) < d-i$. Let $\eta \in Y$ be the generic point of $\mathrm{Supp}(R^if_*\mathcal {O}_X)$. Localizing and using the formal function theorem, we see that

$$\begin{aligned} (R^if_*\mathcal {O}_X)^{\wedge }_{\eta } = \lim _{n \rightarrow \infty } H^i(X_n,\mathcal {O}_{X_n}), \end{aligned}$$

for $X_n = Y \times _X {{\,\mathrm{Spec}\,}}(\mathcal {O}_{X,\eta }/{\mathfrak {m}}^{n+1}_{\eta })$ where ${\mathfrak {m}}_\eta \subset \mathcal {O}_{X,\eta }$ is the maxima ideall. This implies that $i \le \dim X_n = \dim X_0$. Moreover, since $\eta$ is contained in the image of the exceptional set of *f* we see that $\dim \mathrm{Supp}(R^if_*\mathcal {O}_X) + \dim X_0 < d$. Combining those inequalities we obtain the claim.

We now proceed to the proof concerning $R^if_*W\mathcal {O}_{X,\mathbb {Q}}$. Set $S_i = \mathrm{Supp} (R^if_*\mathcal {O}_X)$ We begin by observing that a simple inductive argument with respect to the parameter *n* coming from the exact sequence:

$$\begin{aligned} \cdots \longrightarrow R^if_*\mathcal {O}_X \xrightarrow {V^n} R^if_*W_{n+1}\mathcal {O}_X \longrightarrow R^if_*W_n\mathcal {O}_X \longrightarrow \cdots , \end{aligned}$$

implies that $\mathrm{Supp} (R^if_*W_n\mathcal {O}_X) \subset S_i$. Moreover, from the exact sequence

$$\begin{aligned} \cdots \longrightarrow R^{i-1}f_*W_{n+1}\mathcal {O}_X \xrightarrow {V} R^{i-1}f_*W_n\mathcal {O}_X \longrightarrow R^if_*\mathcal {O}_X \longrightarrow \cdots , \end{aligned}$$

we see that the maps $R^{i-1}f_*W_{n+1}\mathcal {O}_X \longrightarrow R^{i-1}f_*W_n\mathcal {O}_X$ are surjective on $X \setminus S_i$. Consequently, the associated projective system satisfies Mittag-Leffler condition along $X \setminus S_i$ and hence

$$\begin{aligned} \mathrm{Supp}(R^1\lim R^{i-1}f_*W_n\mathcal {O}_X) \subset S_i. \end{aligned}$$

Combination of the above two observations with Proposition 3.4 finishes the proof. $\square$

### Definition 4.5

We say that a morphism of normal varieties $X \rightarrow Y$ is *quasi-birational* if it is surjective purely inseparable and generically finite. Alternatively, taking Stein factorization, it is a composition of a universal homeomorphism and a birational morphism.

### Lemma 4.6

Let $f :X' \rightarrow X$ be a quasi-birational morphism of normal varieties of dimension *d*. Then the map $f^* :H^d(X,W\mathcal {O}_{X,\mathbb {Q}}) \rightarrow H^d(X',W\mathcal {O}_{X',\mathbb {Q}})$ is surjective.

### Proof

First, taking Stein factorization and using the second assertion of Lemma 4.3, we see that we may assume that *f* is birational. In this situation, we conclude by Leray spectral sequence and Lemma 4.4. More precisely, we observe that the top diagonal of the $E_2$ page of the spectral sequence consists of a single entry $H^d(X,W\mathcal {O}_{X,\mathbb {Q}})$ because $f_*W\mathcal {O}_{X',\mathbb {Q}} = W\mathcal {O}_{X,\mathbb {Q}}$ since *X* and $X'$ are normal. This clearly yields the result. $\square$

### Proposition 4.7

Let *X* be a $W\mathcal {O}$-rational proper normal uniruled variety of dimension $d = \dim X$ defined over an algebraically closed field of characteristic $p>0$. Then $H^d(X,W\mathcal {O}_{X,\mathbb {Q}}) = 0$ and $H^d_{{\mathrm{K}\acute{\mathrm{e}}\mathrm{t}}}(X,\mathbb {Q}_p) = 0$.

### Proof

Since *X* is uniruled there exists a generically finite rational map $g :Z \times \mathbb {P}^1 \dashrightarrow X$. Let *Y* be a quasi-resolution of the closure of the graph $\Gamma _g \subset Z \times \mathbb {P}^1 \times X$. The projections induce two maps $Y \rightarrow X$ and $Y \rightarrow Z \times \mathbb {P}^1$ which are proper generically finite and birational, respectively. The map $Y \rightarrow X$ is now only generically finite and therefore we cannot directly compare the Witt vector cohomology of *X* and *Y* by invoking Lemma 4.3.Instead, we proceed by taking the flattening of the morphism $Y \rightarrow X$ (see [40, I.5.2.2]), that is, a proper birational morphism $X' \rightarrow X$ such that the strict transform $Y'$ of *Y* in $X' \times _X Y$ is flat over $X'$. The map $X' \rightarrow Y'$ is flat and generically finite, and hence quasi-finite. It is also proper and therefore finite by [21, Théorème 8.11.1]. Potentially substituting $X'$ by its quasi-resolution and $Y'$ by the normalization of the appropriate pullback, we obtain a finite morphism $Y'\rightarrow X'$ with $Y'$ normal and quasi-birational to $Z \times \mathbb {P}^1$ and $X'$ a quasi-resolution of *X*.

We now show that $H^d(X',W\mathcal {O}_{X',\mathbb {Q}}) = 0$. For this purpose, we first observe that by Proposition 4.6 there exists a surjective morphism $H^d(Z \times \mathbb {P}^1,W\mathcal {O}_{Z \times \mathbb {P}^1,\mathbb {Q}}) \rightarrow H^d(Y',W\mathcal {O}_{Y',\mathbb {Q}})$. However $H^d(Z \times \mathbb {P}^1,\mathscr {O}_{Z \times \mathbb {P}^1}) = 0$ and therefore $H^d(Z \times \mathbb {P}^1,W\mathcal {O}_{Z \times \mathbb {P}^1,\mathbb {Q}}) = 0$ using Remark 4.2, which in turn implies that $H^d(Y',W\mathcal {O}_{Y',\mathbb {Q}}) = 0$. We conclude by using second assertion of Lemma 4.3 for the finite map $Y' \rightarrow X'$.

We finish the proof of the result concerning $W\mathcal {O}$-cohomology, by observing that *X* is $W\mathcal {O}$-rational and hence $H^d(X,W\mathcal {O}_{X,\mathbb {Q}}) = H^d(X',W\mathcal {O}_{X',\mathbb {Q}}) = 0$. The claim about *p*-adic cohomology is now a direct consequence of Corollary 3.18.$\square$

## Proof of Theorem 1.1

In the following section, we prove our main result stating that weakly ordinary varieties with trivial canonical class are not uniruled. We begin with a few preliminary results.

### Maximal rationally chain connected fibrations

We first recall some basic results concerning maximal rationally chain connected (MRCC for short) fibrations. All the results are well-explained in [27, IV.5].

#### Definition 5.1

(Rationally chain connected fibration) Suppose *X* is a normal scheme. Let $X^{\circ } \subseteq X$ be an open subset, and let $\pi ^{\circ } :X^{\circ } \rightarrow S^{\circ }$ be a morphism.

1. We say that $f^{\circ }$ is a *rationally chain connected fibration* if it is a proper morphism satisfying $f^{\circ }_*\mathscr {O}_{X^{\circ }} = \mathscr {O}_{S^{\circ }}$ such that all the geometric fibres are rationally chain connected.
2. We say that $f^{\circ }$ is a *maximal rationally chain connected* if for every other open $X' \subseteq X$ and a rationally chain connected fibration $f' :X' \rightarrow S'$ there exists a rational map $\pi :S' \dasharrow S^{\circ }$ such that $\pi \circ f' = f^{\circ }$.

#### Theorem 5.2

[27, Theorem IV.5.2 & Complement IV.5.2.1] Let *X* be a normal proper variety. Then a maximal rationally chain connected fibration $f^{\circ } :X^{\circ } \rightarrow S^{\circ }$ exists. Moreover, the fibration of a uniruled variety *X* is non-trivial.

#### Proof

The first claim is proven in *loc. cit*. In [50, Proposition A.4] Tanaka reproves the second claim also stating that it is in fact implicit in [27, Theorem IV.5.2]. $\square$

### Étale fundamental groups

Here we present a few results concerning fundamental groups. We begin with the recollection of the standard corollary of the Artin–Schreier sequence.

#### Lemma 5.3

Let *X* be a proper globally *F*-split variety defined over an algebraically closed field of characteristic $p>0$ such that $H^1(X,\mathscr {O}_X) \ne 0$. Then $H^1_{\acute{e}{t}} (X,\mathbb {F}_p)\ne 0$, and consequently *X* admits a non-trivial $\mathbb {F}_p$-covering.

#### Proof

Applying Artin–Schreier sequence of étale sheaves:

$$\begin{aligned} 0 \longrightarrow \mathbb {F}_p \longrightarrow \mathscr {O}_X \xrightarrow {F - {{\,\mathrm{{id}}\,}}} \mathscr {O}_X \longrightarrow 0, \end{aligned}$$

along with the étale descent, we obtain the long exact sequence of cohomologywhere $F :H^1(X,\mathscr {O}_X) \rightarrow H^1(X,\mathscr {O}_X)$ is the natural *p*-linear map induced by the Frobenius on *X*. Using Fitting decomposition in semi-linear algebra (see [22, §6 i)]), we take a decomposition:

$$\begin{aligned} H^1(X,\mathscr {O}_X) = H^1(X,\mathscr {O}_X)^{\mathrm{ss}} \oplus H^1(X,\mathscr {O}_X)^{\mathrm{nil}}, \end{aligned}$$

into a semi-simple part $H^1(X,\mathscr {O}_X)^{\mathrm{ss}}$ generated by *F*-stable elements (over an algebraically closed field), and nilpotent part $H^1(X,\mathscr {O}_X)^{\mathrm{nil}}$ where *F* is nilpotent. Since *X* is globally *F*-split, the map *F* is is bijective, and hence $H^1(X,\mathscr {O}_X)^{\mathrm{ss}} = H^1(X,\mathscr {O}_X) \ne 0$. Consequently, using the above long exact sequence and the fact that the semi-simple part of $H^1(X,\mathscr {O}_X)$ is generated by *F*-stable elements, we see that $H^1_{{\mathrm{K}\acute{\mathrm{e}}\mathrm{t}}}(X,\mathbb {F}_p) \ne 0$, which implies that *X* admits a non-trivial $\mathbb {F}_p$-covering. $\square$

#### Theorem 5.4

[9, Thèoréme] Let *X* be a proper normal rationally chain connected variety defined over an algebraically closed field of characteristic $p>0$. Then $\pi ^{\acute{e}t}_{1}(X)$ is a finite group.

#### Proof

Since the paper we are referencing was unfortunately never published, we provide a sketch of the proof (however following the approach in [9, Théorème] closely). First, we note that for a variety *X* being rationally chain connected is equivalent to existence of:

- closed subvarieties $\{x\} = V_0, V_1,\ldots , V_n = X$, and
- morphisms $\pi _i :T_i \times \mathbb {P}^1 \rightarrow X$, for $i=1,\ldots , n$ such that $\overline{\pi _i(T_i \times \{0\})} = V_{i-1}$ and $\overline{\pi _i(T_i \times \{\infty \})} = V_i$, where $\overline{V}$ denotes the Zariski closure of a subset $V \subset X$.

We can then form a sequence of diagrams of fundamental groups:We now recall that by [12, Lemme 4.4.17] for every dominant morphism of varieties $f :U \rightarrow V$ the image $f_*(\pi ^{\acute{e}t}_{1}(U)) \subset \pi ^{\acute{e}t}_{1}(V)$ is of finite index. Using this result along with the diagrams we see that images of $\pi ^{\acute{e}t}_{1}(V_{i-1})$ and $\pi ^{\acute{e}t}_{1}(V_i)$ are commensurable in $\pi ^{\acute{e}t}_{1}(X)$ and hence one is of finite index if and only if the other is. But $\pi ^{\acute{e}t}_{1}(V_0) = \{1\}$ and $\pi ^{\acute{e}t}_{1}(V_n) = \pi ^{\acute{e}t}_{1}(X)$, and hence the claim follows. We remark that the proof of [12, Lemme 4.4.17] is stated over $\mathbb {C}$, but it actually works verbatim the same over an arbitrary field. $\square$

#### Remark 5.5

In the reference applied in Theorem 5.4, the author also gives a short indication that some of the techniques included in his paper [10] could lead to a slightly more detailed result, potentially useful for our purposes, that the order of the fundamental group is in fact prime to *p*. We were not able to fully verify the claim so we only apply the above result, which is in the end sufficient for our main argument.

### Main result

We now approach the main theorem of the present paper.

#### Theorem 5.6

Let *X* be a normal globally *F*-split and W$\mathscr {O}$-rational proper variety defined over *k*. Suppose that either:

1. *X* satisfies Serre’s condition $S_3$ and $K_X \sim 0$, or
2. *X* is smooth and $K_X$ is numerically trivial.

Then *X* is not geometrically uniruled.

#### Proof

We first make a straightforward reduction of (2) to (1). Indeed, by the standard bijection between *F*-splittings and appropriate sections of $(1-p)K_X$ (see, e.g., [35, Proposition 6] or [48, 4.2]), we have a linear equivalence $(1-p)K_X \sim 0$ since $K_X$ is numerically trivial. Consequently, there exists a variety $X'$ admitting an étale $\mathbb {Z}/(p-1)$-cyclic covering $\varphi : X' \rightarrow X$ trivializing $K_X$, which we claim satisfies all the assumptions of (1). Indeed, $K_{X'} = \pi ^*K_X = 0$ by definition, $X'$ is smooth and hence both normal and $S_3$. Moreover, $X'$ is globally *F*-split, where the splitting of $\mathcal {O}_{X'} \rightarrow F_* \mathcal {O}_{X'}$ is furnished by the pullback via $\varphi$ of the splitting of $\mathcal {O}_X \rightarrow F_* \mathcal {O}_X$.

We now proceed to the proof of (1). We suppose that *X* is a geometrically uniruled variety of dimension *d*, and we seek a contradiction. Taking a separable extension of the base field we may assume that *k* is algebraically closed, *X* is uniruled and the properties of being normal, $S_3$, W$\mathcal {O}$-rational and *F*-split are preserved. Indeed, we use the following references: [49, Tag 0C3M] for normality, Proposition 2.5 for property $S_3$, Proposition 3.12 for W$\mathscr {O}$-rationality and [17, Corollary 2.5] for *F*-splitting.

By Theorem 5.2 there exists an open subset $X^{\circ } \subseteq X$ and a non-trivial MRCC fibration $f^{\circ } :X^{\circ } \rightarrow S^{\circ }$. We claim that *all the stated properties of* *X*, *that is normality, property* $S_3$, *F-splitting and triviality of the canonical bundle except possibly* $W \mathcal {O}$*-rationality, are inherited by the geometric generic fibre* $X_{\overline{\eta }}$* of* $f^{\circ }$. Indeed, by Corollary 2.3, $X_{\overline{\eta }}$ is globally *F*-split and normal. Then Proposition 2.5 directly implies that the geometric generic fibre is $S_3$. Other properties follow from adjunction, which concludes the proof of our claim.

By the definition of the MRCC fibration, we know that $X_{\overline{\eta }}$ is rationally chain connected. Furthermore, by Proposition 3.22, $X_{\overline{\eta }}$ admits a $\mathbb {Q}_p$-rational quasi-resolution. Consequently, by replacing *X* be $X_{\overline{\eta }}$, we may assume that:

1. *X* is normal and $S_3$,
2. *X* is globally *F*-split,
3. $$K_X$$ is trivial,
4. *X* is rationally chain connected, and
5. *X* admits a $\mathbb {Q}_p$-rational quasi-resolution.

We claim that we can also assume that $H^1(X,\mathscr {O}_{X}) = 0$. Indeed, we first observe that all the above properties are preserved by étale covers. For (1)–(4) we use the arguments given above, and (5) follows from Lemma 3.7 combined with Proposition 3.16. We now use Theorem 5.4 to see that the étale fundamental group of *X* is finite, and hence we may substitute *X* with its universal cover $X'$. It satisfies all the properties and the vanishing $H^1(X,\mathcal {O}_{X'}) = 0$ by Lemma 5.3.

Equipped with the above, we now use Proposition 2.4 (*X* is $S_3$) in order to see that $H^{d-1}(X,\mathscr {O}_X)$ vanishes too. Consequently, Proposition 4.1 gives non-vanishings $H^d_{{\mathrm{K}\acute{\mathrm{e}}\mathrm{t}}}(X,\mathbb {Q}_p) \ne 0$. We now consider the $\mathbb {Q}_p$-rational quasi-resolution $Y \rightarrow X$ whose existence is postulated above. By $\mathbb {Q}_p$-rationality and the Leray spectral sequence for *p*-adic cohomology (see Proposition 3.15) we know thatSince $Y \rightarrow X$ is quasi-birational it is by definition generically a universal homeomorphism, and hence using [28, 6.6 Proposition] there exists a rational map $X \dasharrow Y$. Since *X* is uniruled this implies that *Y* is uniruled too. Consequently the non-vanishing above violates Proposition 4.7 yielding the desired contradiction. The proof is thus finished. $\square$

Using the results of Langer [29, Corollary 3.3], we obtain the following corollary.

#### Corollary 5.7

Let *X* be a weakly ordinary projective variety with trivial canonical class. Then the tangent bundle $\mathscr {T}_X$ is strongly semistable with respect to every polarization.

We now provide three classes of examples justifying certain steps in the above reasoning.

#### Remark 5.8

We attempted to prove the above theorem in the more general setting where *X* is strongly *F*-regular and $K_X$ is only numerically trivial. Under these assumptions the $\mathbb {Z}/(p-1)$-cyclic covering trivializing $K_X$ appearing in the proof is only quasi-étale. Unfortunately, neither W$\mathscr {O}$-rationality nor rational chain connectedness is preserved under quasi-étale maps without any additional assumptions. For the statement concerning rational chain connectedness, one considers the following example. Let $N \in \mathbb {N}$ be coprime to *p* and . Let *C* be a smooth projective curve of positive genus defined over an algebraically closed field and equipped with a *G*-action such that $C/G \simeq \mathbb {P}^1$. Moreover, assume that *G* acts linearly on $\mathbb {P}^2$ via diagonal matrices $\mathrm{diag}(\xi ^a,\xi ^b,\xi ^c)$, where $\xi$ is the *N*-th primitive root of unity and the numbers *a*, *b*, *c* and *N* are pairwise coprime (so that the action is free in codimension one). The variety $\mathbb {P}^2 \times C$ is clearly not rationally chain connected. We claim that the quotient $(\mathbb {P}^2 \times C)/ G$ is rationally chain connected and the map $\mathbb {P}^2 \times C \rightarrow (\mathbb {P}^2 \times C) / G$ is quasi-étale. The second claim is clear, because the fixed points of the action on the product occur in codimension two. For the first, we observe that the natural map

$$\begin{aligned} (\mathbb {P}^2 \times C)/G \rightarrow C/G = \mathbb {P}^1 \end{aligned}$$

is generically a $\mathbb {P}^2$-bundle over $\mathbb {P}^1$ admitting a section by Tsen’s theorem stating that the Brauer group of a function field of a curve over an algebraically closed field is trivial and hence all $\mathbb {P}^n$-bundles admit a section (see [49, Tag 03RC]). This example also shows that fundamental groups behave wildly under quasi-étale morphisms. For example, we see that $\pi _1\left( (\mathbb {P}^2 \times C)/G\right)$ is finite, and $\pi _1(\mathbb {P}^2 \times C) \simeq \pi _1(C)$ is infinite highly non-commutative.

#### Example 5.9

One could hope that the non-vanishing of top rational Witt vector cohomology holds for every weakly ordinary Calabi–Yau variety. Unfortunately, this is not the case. We claim that the counterexample is provided by singular Enriques surfaces in characteristic two. Indeed, from the Bombieri–Mumford classification (see [32, Section 1] for the details) we know that for such a surface *S* the canonical divisor is trivial and moreover the vector spaces $H^1(S,\mathscr {O}_S)$ and $H^2(S,\mathscr {O}_S)$ are one-dimensional with bijective Frobenius action. This implies that such *S* is weakly ordinary and Calabi–Yau. However, in [23, II, Proposition 7.3.2], Illusie proves that the top Witt vector cohomology $H^2(S,W\mathscr {O}_S)$ is 2-torsion, and hence vanishes after tensoring with $\mathbb {Q}$. It turns out (see [30]) that in dimension two such examples arise only in characteristic two and three. Unfortunately, we do not know if similar examples exist in higher dimensions.

#### Example 5.10

We now present an example of a variety which is of trivial canonical class, Gorenstein, normal, globally *F*-split and rational but fails to admit $\mathbb {Q}_p$-rational quasi-resolution. It furnishes a counterexample to our main statement Theorem 5.6 if the W$\mathcal {O}$-rationality assumption is dropped. Luckily we managed to rule it out as a general fibre of an MRCC fibration. Let $E \subset \mathbb {P}^2$ be a globally *F*-split (equiv. ordinary) elliptic curve. We consider the pair $(\mathbb {P}^2,E)$ which is log Calabi–Yau, that is, the divisor $K_{\mathbb {P}^2} + E$ is linearly equivalent to zero. Moreover, by [43, Proposition 7.2], we know that the pair is also globally *F*-split. Taking a blow-up *X* of $\mathbb {P}^2$ in ten points $P_i$ lying on *E* we obtain a morphism of pairs $f :(X,{\tilde{E}}) \rightarrow (\mathbb {P}^2,E)$, where ${\tilde{E}}$ is the strict transform of *E*. Since the points lie on the curve the morphism is crepant, and therefore the pair $(X,{\tilde{E}})$ is also globally *F*-split and log Calabi–Yau. Let $L_i$ be the exceptional curve over $P_i$. The normal bundle of ${\tilde{E}}$ is anti-ample, and therefore there is hope for contracting it. This can actually be performed using Keel’s technique (see [26]) for the big and nef divisor

$$\begin{aligned} 2{\tilde{E}} + (L_1 + L_2), \end{aligned}$$

assuming that

5.10.a $$\begin{aligned} \left. 2{\tilde{E}} + (L_0 + L_1) \right| _{\tilde{E}} \sim 2E|_E - 2\left( \sum _i P_i \right) + (P_1 + P_2) \end{aligned}$$

is a torsion divisor on $E \cong \tilde{E}$. The points $P_i$ can always be chosen so that the divisor of (5.10.a) is torsion. So, let us assume that we made such a choice, and let *Y* be the surface obtained by contracting $\tilde{E}$. We claim that *Y* *satisfies our requirements—it is Gorenstein, normal, globally* *F-split and rational*. The canonical divisor $K_Y$ is the pushforward of $K_X + {\tilde{E}}$ and hence is trivial. This implies that *Y* is Gorenstein and normal. The necessary *F*-splitting comes from a pushforward of a global *F*-splitting on *X*.

The variety *Y* is also interesting cohomologically. More precisely, using proper base change and the fact that *E* is ordinary we may prove that the natural resolution $X \rightarrow Y$ is not $\mathbb {Q}_p$-rational. Then, the same holds for any other resolution too, because $X \rightarrow Y$ is the minimal resolution. Moreover, using Leray spectral sequence for W$\mathscr {O}$-cohomology for the same resolution $f :X \rightarrow Y$ we obtain the following diagram describing $E_2$-page of the spectral sequence (we note that $f_*W\mathscr {O}_{X,\mathbb {Q}} = W\mathscr {O}_{Y,\mathbb {Q}}$): which yields an isomorphism $H^2\left( Y,W\mathscr {O}_{Y,\mathbb {Q}}\right) \simeq H^1\left( E,W\mathscr {O}_{E,\mathbb {Q}}\right)$ since $H^1\left( X,W\mathscr {O}_{X,\mathbb {Q}} \right) =H^2\left( X,W\mathscr {O}_{X,\mathbb {Q}} \right) = 0$ using rational chain connectedness. As *E* is ordinary, $0 \ne H^1\left( E,W\mathscr {O}_{E,\mathbb {Q}}\right) \cong H^1_{{\mathrm{K}\acute{\mathrm{e}}\mathrm{t}}}(E,\mathbb {Q}_p) \otimes _{\mathbb {Q}_p} K$, and hence Proposition 4.7 does not hold true without appropriate singularity assumptions.
